# 3D-printed hydrogel particles containing PRP laden with TDSCs promote tendon repair in a rat model of tendinopathy

**DOI:** 10.1186/s12951-023-01892-5

**Published:** 2023-06-03

**Authors:** Congsun Li, Jie Wang, Weinan Yang, Kang Yu, Jianqiao Hong, Xiaoxiao Ji, Minjun Yao, Sihao Li, Jinwei Lu, Yazhou Chen, Shigui Yan, Haobo Wu, Chiyuan Ma, Xiaohua Yu, Guangyao Jiang, An Liu

**Affiliations:** 1 Department of Orthopedic Surgery, The Second Affiliated Hospital, Zhejiang University School of Medicine, Hangzhou City, Zhejiang Province PR China; 2 Orthopedics Research Institute of Zhejiang University, Hangzhou City, Zhejiang Province PR China; 3 Key Laboratory of Motor System Disease Research and Precision Therapy of Zhejiang Province, Hangzhou City, Zhejiang Province PR China; 4 Clinical Research Center of Motor System Disease of Zhejiang Province, Hangzhou, PR China; 5 State Key Laboratory of Fluid Power and Mechatronic Systems, School of Mechanical Engineering, Hangzhou, Zhejiang PR China

**Keywords:** Hydrogel, TDSCs, Tendinopathy, Inflammation, PI3K-AKT pathway

## Abstract

Long-term chronic inflammation after Achilles tendon injury is critical for tendinopathy. Platelet-rich plasma (PRP) injection, which is a common method for treating tendinopathy, has positive effects on tendon repair. In addition, tendon-derived stem cells (TDSCs), which are stem cells located in tendons, play a major role in maintaining tissue homeostasis and postinjury repair. In this study, injectable gelatine methacryloyl (GelMA) microparticles containing PRP laden with TDSCs (PRP–TDSC–GM) were prepared by a projection-based 3D bioprinting technique. Our results showed that PRP–TDSC–GM could promote tendon differentiation in TDSCs and reduce the inflammatory response by downregulating the PI3K–AKT pathway, thus promoting the structural and functional repair of tendons in vivo.

**Graphical Abstract:**

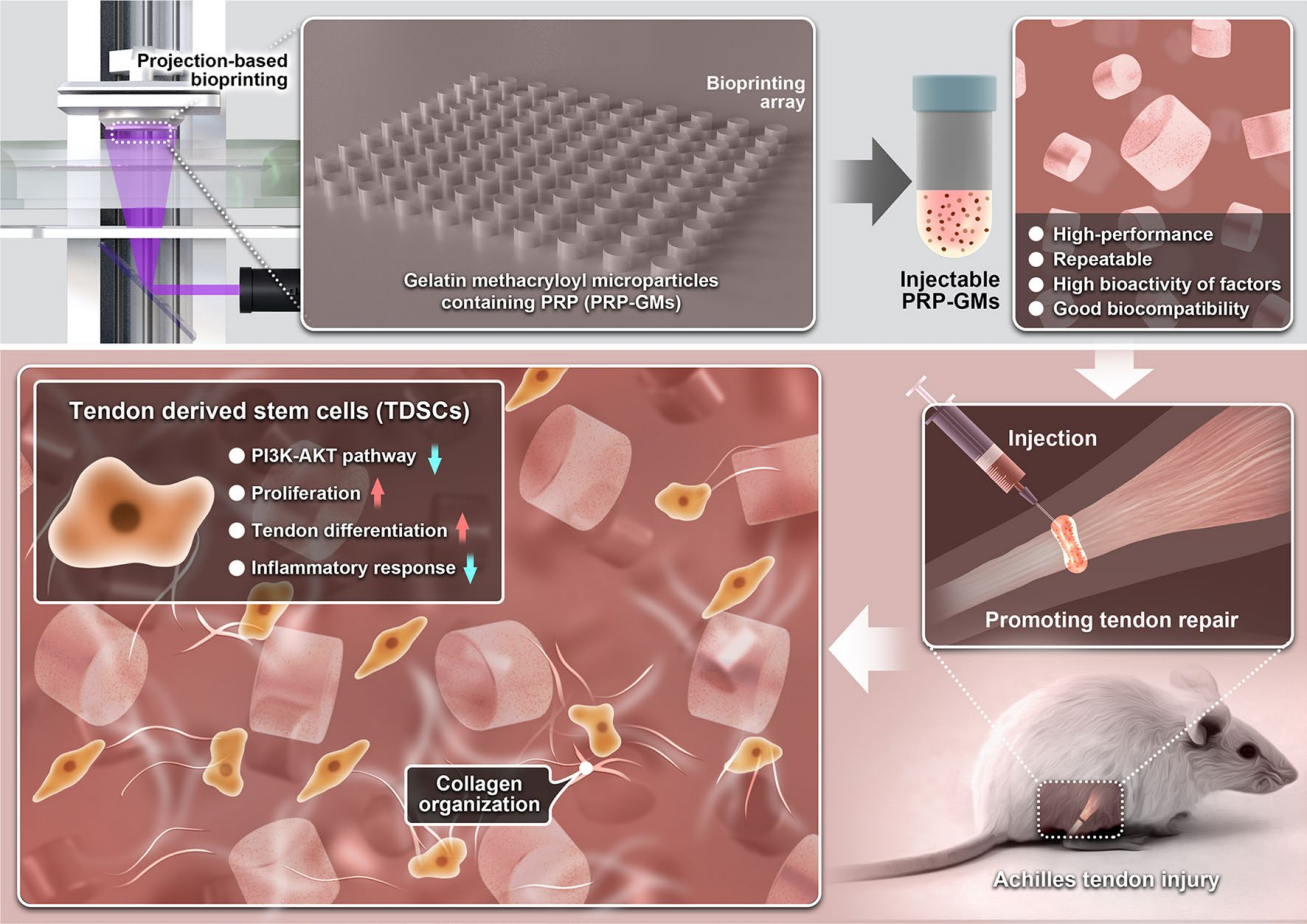

**Supplementary Information:**

The online version contains supplementary material available at 10.1186/s12951-023-01892-5.

## Introduction

Achilles tendon injury is a common motor system disease. In the early stage of acute injury, a series of reactions initiate repair, including but not limited to inflammatory reactions and the haematoma state [1–3]. However, persistent inflammation can lead to chronic tendinopathy, which exacerbates pathological processes and increases the complexity of dysfunction and rehabilitation management [4]. Tendon injury lasting more than 4 weeks is defined as chronic tendon injury [5], which typically leads to heterotopic ossification, persistent pain, and potential re-rupture. Patients with chronic tendinopathy are in a chronic state of low-grade inflammation, which may be a risk factor for failure of the healing response after acute tendon injury, making it easier for patients to have disrupted healing even after surgery [2, 6]. Tendinopathy restricts normal activities and has continuous physical and psychological effects [7, 8]. At present, treatments for tendinopathy include surgical treatment, blocking therapy, shock wave therapy, nonsteroidal drug therapy and PRP injection therapy [9]. Among the nonoperative treatments, PRP injection has received much attention and been widely used in recent years. The recent reviews of PRP therapy show its positive role in treating tendinopathy [10–12] due to being rich in bioactive cytokines such as HGF, VEGF, TGF- β and FGF [13, 14]. Some studies have shown that leukocyte-poor PRP combined with BMSCs or ADSCs can relieve tendon pain and improve functional outcomes [15–17]. However, once the concentration of PRP exceeds a certain limit, cell proliferation and growth are inhibited [14]. Typically, PRP therapy requires several courses to ensure its therapeutic effect in the clinic. In addition, mesenchymal stem cell therapy has recently been proposed for treating musculoskeletal diseases, including tendinopathy [18–20]. As stem cells located in tendons, TDSCs retain more tendon-specific differentiation properties in addition to their stem cell properties, which may give them an advantage over BMSCs for musculoskeletal tissue engineering [21–23]. The migration and differentiation of tendon stem cells are significant in tendon repair [24], but low cell retention after direct in situ injection is still the main obstacle to the clinical application of stem cell transplantation [25]. Therefore, this study aimed to design a PRP delivery system loaded with TDSCs for the treatment of tendinopathy, and hydrogel particles meet this demand.

Methacrylic acid gelatine (GelMA) is a kind of photopolymerizable biomaterial derived from gelatine that possesses similar properties to the natural extracellular matrix and is suitable for cell adhesion. Due to its robust biocompatibility and adjustable physical properties, it is widely used to print biological tissue engineering scaffolds [26] to provide three-dimensional support for cell growth and tissue repair [27].

At present, methods such as emulsification[28] and microfluidic chips [29, 30] have been used to manufacture microgels. However, the toxic substances in the production process and the high cost still obstruct their development. In this study, PRP-TDSC-GM was prepared by a projection-based 3D bioprinting technique and injected into a rat model of tendinopathy to evaluate their effect on the repair of tendon structure and function under chronic inflammatory conditions.

## Materials and results

### PRP and GelMA hydrogel preparation

As previously described [31], PRP was prepared from whole blood under aseptic conditions. Briefly, blood was collected in an EDTA-K2 sodium container by cardiac puncture from healthy SD rats. The blood was centrifuged at 200 ×*g* for 10 min, and the upper leukocyte/platelet layer and plasma layer were transferred to a new 15 ml centrifuge tube. After centrifugation at 180× g for 10 min to remove leukocytes, the upper layer was collected and centrifuged at 600 ×*g* for another 10 min to enrich platelets. The platelet mass was resuspended to obtain an appropriate concentration of PRP and was stored at − 80 °C until use. To determine PRP quality, each sample was subjected to a component test via an automatic haematology analyser system (BC-5300; Mindray). GelMA and the photoinitiator phenyl-2-pyrrol-6-trimethylbenzoyl-phosphonate (LAP, 0.05% w/v, GelMA 10% w/v, EFL-GM-1 M, Yongqinquan Intelligent Equipment Co., Ltd, Suzhou, China) were suspended in PBS. Next, the solution was incubated in a water bath at 50 °C until completely dissolved. Finally, the solution was filtered with a 0.22-µm filter and then stored at 4 °C away from light.

### Biofabrication and characteristics of PRP-GM

GM and PRP-GM was fabricated with a projection-based 3D bioprinting system (Suzhou Intelligent Manufacturing Institution, China). Due to the projection-based printing mechanism, GM was printed with high consistency and efficiency. Specifically, GM was designed into particle arrays with Solidworks (version 2016) and then exported as an STL file before being imported into the printing software. According to previous work, the printing parameters were set at 48 s for each layer, and the layer thickness was 200 μm. When the printing begins, the deposition platform drops to a height one layer away from the bottom of the trough. The light starts, and the digital micromirror device (DMD) shows the slice mass before the light is reflected to the bottom of the material trough. After 48 s of exposure (405 nm, 11 mW/cm^2^), the deposition platform is lifted after the GelMA or PRP-GelMA is ossified, and printing is completed. By adjusting the amount of PRP added, GM containing different concentrations of PRP (0%, 10%, 20%, and 40% w/w) were produced and used to perform subsequent studies.

### In vitro enzymatic degradation of GM

To characterize the degradation properties of GelMA microgels, 200-µl hydrogel samples were placed in 1 ml centrifuge tubes with 1 ml of PBS containing 1–2 U ml^–1^ type II collagenase at 37 ℃ [32]. At a predefined time, the collagenase solution was removed to end the digestion, and the GM was observed under a microscope.

### Extraction and identification of TDSCs

Four male SD rats weighing 200–250 g were deeply anaesthetized with pentobarbital, and the lower limbs were disinfected. The middle part of the Achilles tendon was cut and immersed in a sterile phosphate buffered saline solution (PBS) containing high concentrations of bicarbonate (penicillin 500 µg/mL, streptomycin 1000 µg/mL) for 5 min and then washed twice with PBS without antibiotics. The tendon tissues were chopped and then digested with type I collagenase on a shaking bed at 37 °C for 4 h. The undigested tissue residue was removed through a 10 μm cell filter. The cells were washed twice with PBS and then suspended in modified low-glucose DMEM containing 10% foetal bovine serum. The cells were placed in an incubator at 37 °C and 5% CO_2_ overnight to form colonies. On Day 2, the cells were washed with PBS to remove nonadherent cells. The medium was changed every two days, and after three generations, the cells were used for follow-up experiments. Flow cytometry was used to identify tendon stem cells, and the markers were CD31, CD106, CD44 and CD90.

### Cell proliferation, migration, and morphology in response to PRP-GM treatment

TDSCs were inoculated in 6-well plates (1 × 10^− 5^/well) and cultured in Dulbecco’s modified Eagle’s medium (DMEM) under different conditions: (a) NC group (no stimulation); (b) IL-1 group (IL-1:10 ng/ml); (c) GM group (IL-1:10 ng/ml and 200 µl of GM); (d) 20% PRP-GM group (IL-1:10 ng/ml and 200 µl of 20% PRP-GM); and (e) PRP group (IL-1: 10 ng/ml & 40 µl of PRP). Cell morphology was observed under a microscope on the 7th day. To examine cell proliferation, GM was first added to the wells of a 96-well cell culture plate. Then, TDSCs in good growth conditions were prepared as a cell suspension of 5 × 10 ^− 4^ cells/mL, 100 µl of which was added to each well. On Days 1, 4, and 7, the original medium was removed, and fresh medium containing 10% CCK-8 solution (v/v) was added to each well. After 2 h of culture without light, the absorbance at 450 nm was measured (Spectra Max 190, USA). After the baseline reading of each group was subtracted, the result was expressed as light absorbance (OD value). The proliferation of TDSCs treated with different PRP-GM was evaluated in the same way.

Wound healing and Transwell assays were used to examine the effect of PRP-GM on TDSC migration. Cells were seeded at a density of 3 × 10^4^ cells/well in the Ibidi Culture-Insert 2 Well (Cat. No: 81,176 Ibidi Company) for the wound healing assay. The following groups were used: (a) NC group (no stimulation); (b) IL-1 group (IL-1:10 ng/ml); (c) GM group (IL-1:10 ng/ml and 200 µl of GM); (d) 10% PRP-GM group (IL-1:10 ng/ml and 200 µl of 10% PRP-GM); (e) 20% PRP-GM group (IL-1: 10 ng/ml and 200 µl of 20% PRP-GM); and (f) 40% PRP-GM group (IL-1: 10 ng/ml and 200 µl of 40% PRP-GM). Then, images of the cells were captured with a microscope (Olympus) after 24 and 48 h of culture. Finally, the ratio of the blank area at 24 and 48 h to that at 0 h in each group was quantitatively analysed by ImageJ software. In addition, specific 24-well plates (8 μm) were used for the Transwell test. Approximately 5 × 10^− 3^ cells were inoculated into upper chambers and cultured with PRP-GM for 24 h. After the cells were fixed and stained with crystal violet, the number of migrated cells in each group was counted and used for comparisons.

### Scanning electron microscopy (SEM) and transmission electron microscopy (TEM)

The morphology of GM was observed by SEM. First, the samples were fixed with 2.5% glutaraldehyde, fixed with 1% osmic acid for 1 h and dehydrated with a graded series of ethanol (30–100%). Finally, the samples were subjected to critical point dehydration (Hitachi). After surface spraying gold treatment, the GM was observed by SEM (SU-8010; Hitachi).

Tendon samples were double fixed with glutaraldehyde and osmic acid and then embedded in epoxy resin after being stained with lead citrate and uranyl acetate. Longitudinal and transverse slices were prepared and observed by TEM (Hitachi HT7700, Japan).

### Cell differentiation in response to PRP-GM (histological, immunofluorescence, and immunohistochemical assays and Western blotting)

For the histological assay, the collected specimens were fixed in formalin for 24 h, dehydrated by an alcohol gradient, and embedded in paraffin blocks. Four-micron histological sections were prepared by a slicing mechanism and observed after haematoxylin-eosin (H&E) staining.

For the immunofluorescence assay, histological sections were subjected to antigen retrieval and blocked with 5% BSA. Cells or sections were incubated with anti-COL1A1 (Proteintech, 66761-1), anti-COL3 (Proteintech, 22734-1), anti-TNC (Proteintech, 67710-1), anti-TNMD (Abclonal, A17753), anti-TBP (Proteintech, 66166-1) and anti-SCX (Abcam, ab58655) antibodies. After FITC-phalloidin and DAPI immunofluorescence staining, a high-resolution laser-scanning microscope was used for observation.

For the immunohistochemical assay, after antigen repair, 3% H_2_O_2_ was used to inhibit endogenous peroxidase before 5% BSA blocking treatment. Then, the slices were incubated with primary antibodies and secondary antibodies. The positive reaction was shown with a peroxidase substrate solution, and the section of the reaction turned brown and was further analysed.

Total proteins were extracted from cells with RIPA buffer (Boster, Wuhan, China) containing protease/phosphatase inhibitor cocktails (Boster, Wuhan, China) to avoid protein degradation. After insoluble impurities were removed, the proteins were separated by 10% SDS–PAGE (20 µL/path) and then transferred onto polyvinylidene difluoride (PVDF) membranes. After being blocked with 5% BSA for 10 min, the membranes were incubated with primary antibodies overnight at 4 ℃. Then, the membranes were washed with TBS containing 0.1% Tween-20 and incubated with secondary antibodies for 1 h. The membranes were washed again, and target proteins were detected and visualized by chemiluminescence immunoassays with a Bio-Rad XRS system (Hercules, CA, USA).

### Transcriptome sequencing assay

TDSCs were inoculated in 6-well plates (1 × 10^− 5^/well) and cultured in Dulbecco’s modified Eagle’s medium (DMEM) under different conditions: (a) NC group (no stimulation); (b) IL-1 group (IL-1:10 ng/ml); and (c) 20% PRP-GM group (IL-1:10 ng/ml &100 µl of 20% PRP-GM). On Day 7, total RNA was extracted from TDSCs by TRIZOL reagent (Invitrogen) and reverse transcribed into cDNA by Prime Scrip RT Master Mix (Takara). The samples were then analysed by LianChuan Biotechnology Co., Ltd. (Hangzhou, China).

### Establishment of rat Achilles tendon injury

With the approval of the Institutional Animal Protection and Use Committee, 36 female SD rats (200–250 g) were purchased from SLAC (Shanghai, China) for in vivo research. Once fully exposed and dissociated, the Achilles tendon was cut off horizontally at a distance of 0.5 cm from the calcaneus. The skin was sutured, and the rats were allowed to move freely in captivity [33]. The surgery was performed under general anaesthesia with pentobarbital sodium (36 mg/kg).

Four weeks later, the rats were divided into 3 groups: (a) SHAM group (no treatment); (b) TDSC-GM group (injection of 100 µl of TDSC-GM); and (c) PRP-TDSC-GM group (injection of 100 µl of PRP-TDSC-GM). All rats were sacrificed 4 or 8 weeks after injection, and the tendons were collected for histology, immunohistochemistry, TEM and tension tests.

### Tension test of the tendon

The rats were euthanized under excessive anaesthesia, and then the Achilles tendon was cut one centimetre from the proximal end and peeled off to the attachment point of the metatarsal bone. The tendons were completely removed and wrapped in sterile gauze. Finally, the tendons were soaked in PBS and preserved at − 80 °C before the test. A material test system (Instron 5944) was used to conduct the tendon tension test. The ends of the tendon were fixed on the test system, and the tendon was stretched at a displacement control rate of 100% strain per second until the tendon was broken. The tension value was recorded at this time, which was the maximum load the tendon could bear.

### Statistical analysis

All quantitative results are presented as the mean ± standard deviation (SD). The graphs were visualized using GraphPad Prism 8. Statistical analysis was conducted by ANOVA or two-way ANOVA and are expressed as ^∗^P < 0.05, ^∗∗^P < 0.01, ^∗∗∗^P < 0.001, and ^∗∗∗∗^P < 0.0001.

## Results

### Migration and differentiation of TDSCs treated with PRP-GM

**Fig. 1:**
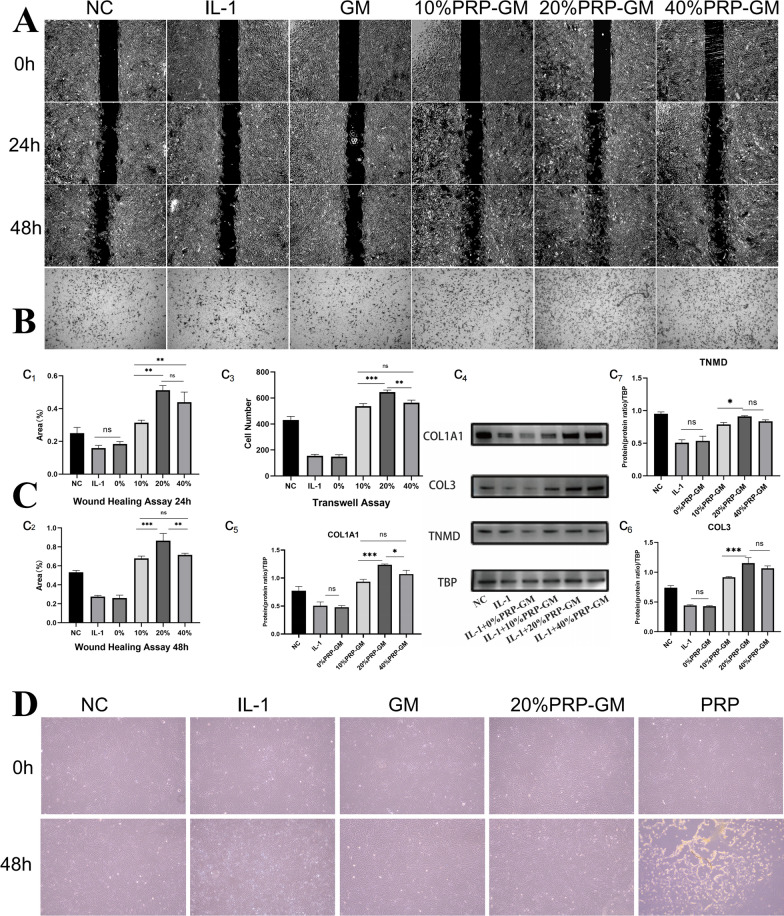
The effects of PRP-GM on migration, proliferation and morphology. **A** Wound healing assay showing that PRP-GM promoted TDSC migration. **B** The migration of TDSCs was evaluated by Transwell assay. **C** (c_1_–c_3_) Quantitative analysis of A and B (n = 3, respectively). (c_4_) Western blot analysis of COL1A1, COL3 and TNMD in TDSCs after tenogenic differentiation for 5 days in the different groups (n = 3). (c_5_–c_7_) Quantitative analysis of c_4_. **D** Morphological changes in TDSCs on Day 5 under different conditions. The data are presented as the mean ± SD. ANOVA was performed for statistical analysis (*p < 0.05, **p < 0.01, ***p < 0.001)

The wound healing assay showed that the migration of TDSCs cultured with 20% PRP-GM was significantly better than that in the other groups at 24 and 48 h, but there was no significant difference between 10% PRP-GM and 40% PRP-GM at 48 h (Fig. 1A, C). In the Transwell assay, 20% PPR-GM exerted the most promotional effects on the migration of TDSCs (Fig. 1B, C). Western blotting indicated that 20% PRP-GM mostly upregulated the expression of COL1A1, COL3, and TNMD on the 7th day compared with that in the other groups (Fig. 1c_4_). Therefore, 20% PRP-GM was selected for subsequent studies. In addition, the wound healing and Transwell assays indicated that IL-1 attenuated the migration of TDSCs.

### Biofabrication, biocompatibility and characterization of GM

**Fig. 2:**
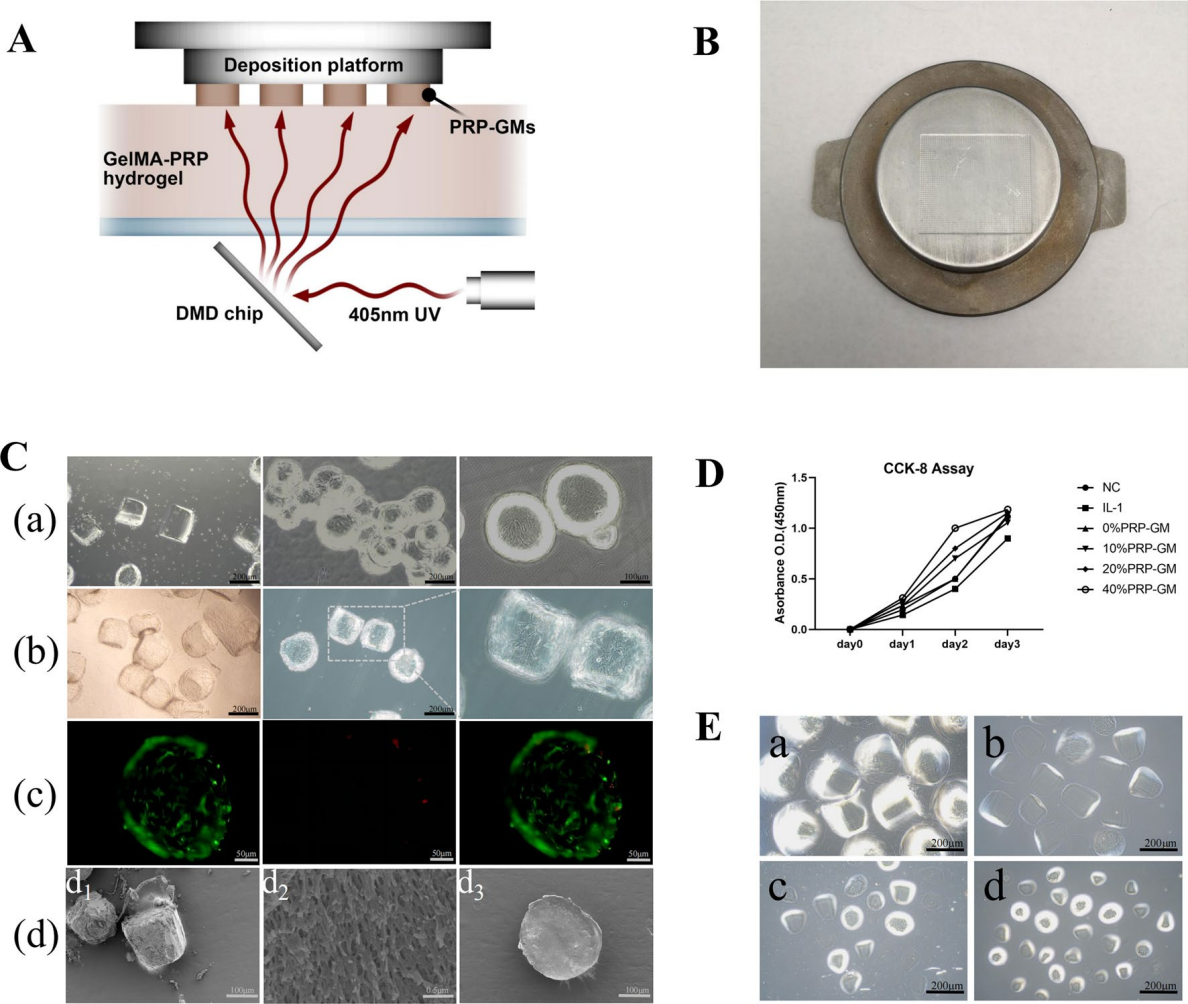
**A** Diagram of the projection-based 3D bioprinting device for the preparation of GM. **B** GM on the printing platform. **C** (a) GM under an optical microscope. (b) Coculture of TDSCs and GM. (c) Immunofluorescence staining of living (green) and dead (red) cells on GM. (d_1_) GM under SEM (× 30k). (d_2_) Surface morphology of GM under SEM(× 30k). (d_3_) Cross-section of GM under SEM (× 30k). **D** Visualization diagram of cell proliferation evaluated by CCK-8 (n = 3). **E** Enzymatic degradation of GM: a: 0 h; b: 2 h; c: 4 h; d: 8 h

The biofabrication process of GM is presented in Fig. 2A and B, and GM showed good size uniformity under a microscope (Fig. 2C). Culturing GM with TDSCs showed great biocompatibility . The results of fluorescent live/dead staining showed that there was no significant difference in the survival rate on the first, forth and seventh day between the GM group and PRP-GM group (Fig. 2C, Supplementary Fig. 1C). CCK-8 analysis showed that compared with that in the normal group, the growth of TDSCs was slowed by IL-1 stimulation, and the addition of PRP-GM could reverse this inhibitory effect and improve cell proliferation ( Fig. 2D). In order to explore the degradability of hydrogel particles, we carried out enzyme degradation experiments in vitro. The experimental results indicated that GM showed good degradation performance at low enzyme concentration (Fig. 2E).

### Effects of PRP-GM on TDSC differentiation

**Fig. 3:**
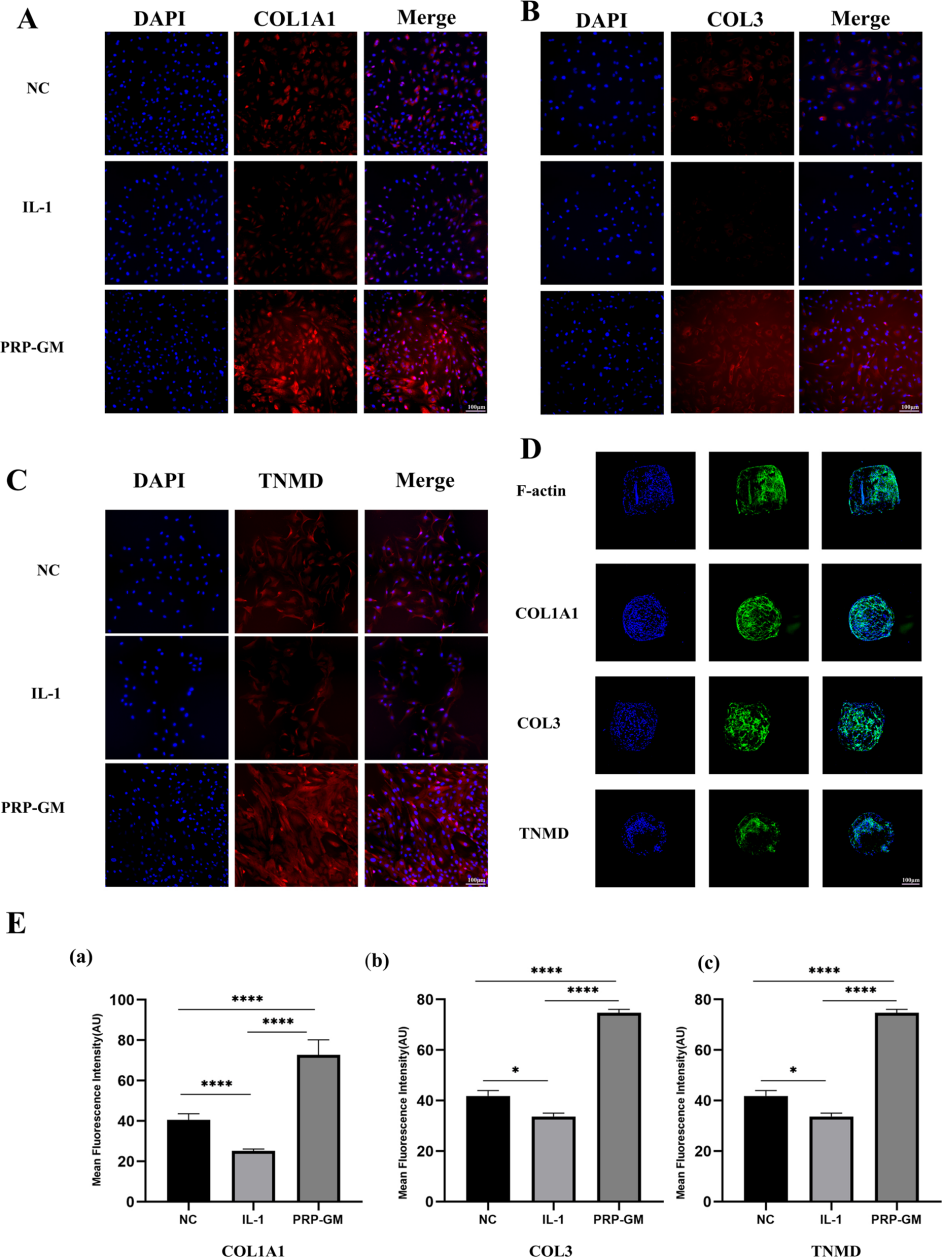
**A**–**C** Immunofluorescence staining showed COL1A1, COL3 and TNMD expression in TDSCs cocultured with different conditions in plates. **D** Protein expression of TDSCs cultured on microgels. **E** (a–c) Quantitative analysis of A, B, and C (n = 3)

GM and TDSCs were cocultured, and in the NC group, the cells were pebble-shaped, which is a typical characteristic of TDSCs. However, TDSCs became longer and slender after PRP-GM treatment, and this effect was more obvious at the cell junctions. In the group treated with pure PRP, TDSCs stopped growing and became sparse (Fig. 1D). Immunofluorescence staining indicated that PRP-GM upregulated the expression of COL1A1, COL3, and TNMD on the 7th day compared with that in the NC and IL-1 groups (Fig. 3A, B, C, E). F-actin, COL1A1, COL3 and TNMD immunofluorescence staining revealed that TDSCs on GM synthesized matrix components on Day 4 (Fig. 3D). The results of Western Blotting after 10 days of culture showed that the expressions of tendon-associated proteins in TDSCs grown on PRP-GM were higher than that on GM (Supplementary Fig. 2B).

### Histological analysis of tendon repair

**Fig. 4:**
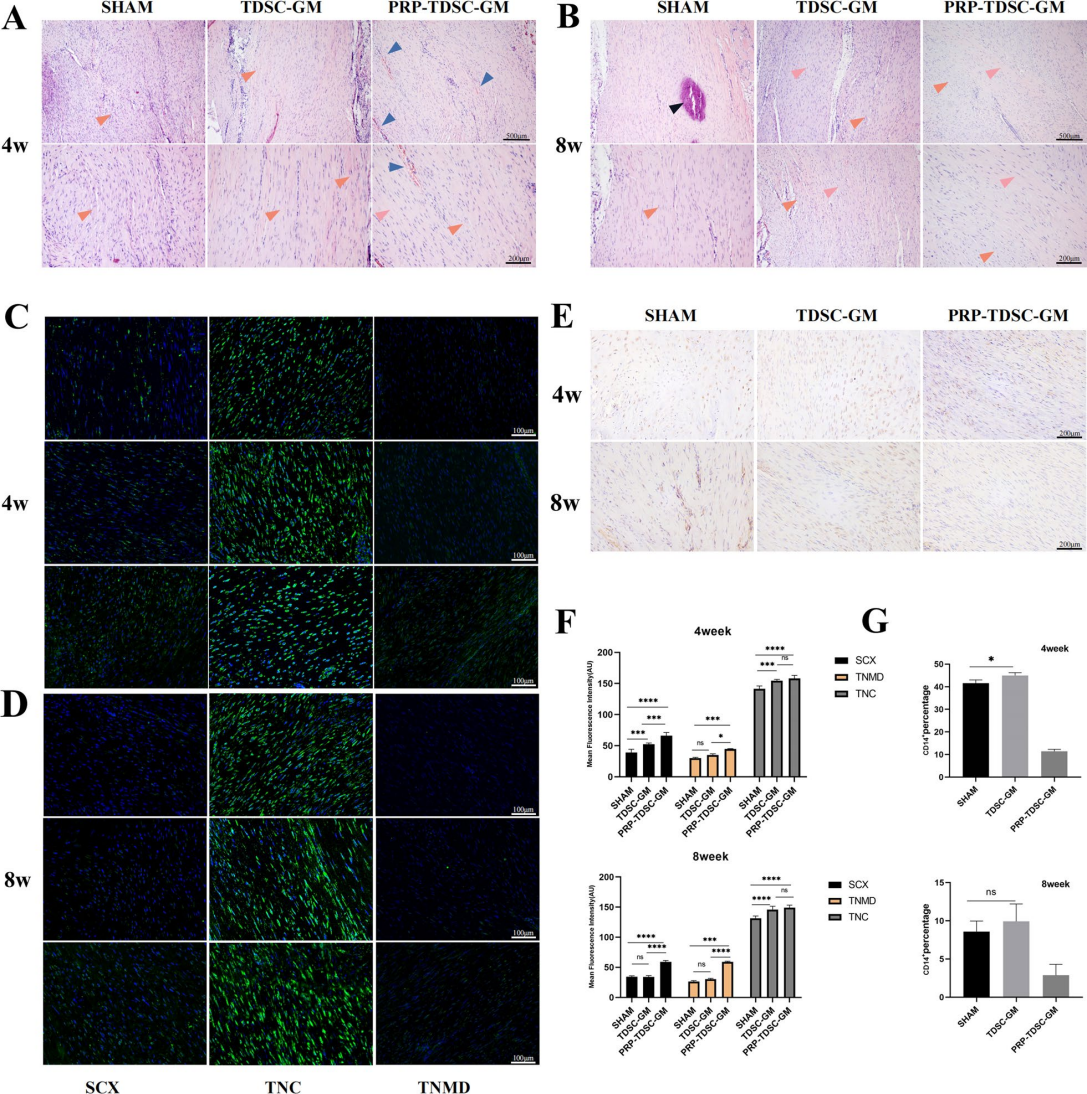
**A**, **B** Representative images of H&E staining of tendon tissues in the different groups at the 4th week and 8th week after treatment. Yellow arrow: immature fibrous tissue. Blue arrow: neovascularization. Pink arrow: mature fibrous tissue Black arrow: calcific foci. **C**, **D** Protein expression of SCX, TNMD and TNC in tendon tissues in the different groups in the 4th week and 8th week after treatment. **E** Immunohistochemical images of CD14^+^ cells **F** Quantitative analysis of **C** and **D**. **G** Quantitative analysis of E(n = 3, *p < 0.05, **p < 0.01, ***p < 0.001)

The effect of tendon repair was evaluated by H&E staining. Noticeably decreased pathological manifestations were observed in the TDSC-GM treatment group and PRP-TDSC-GM treatment group. More specifically, the Achilles tendon tissue samples from the SHAM group were significantly different in appearance, showing the loss of collagen fibrils, disarrangement of collagen fibrils and inflammatory cell infiltration at the injured site. Collagen fibres were arranged more tightly and regularly, and the infiltration of inflammatory cells was decreased in the TDSC-GM and PRP-TDSC-GM groups (Fig. 4A, B). At the 4th week after injection treatment, compared with PRP-TDSC-GM group, tendons are more thicken in both sham group and TDSC-GM group, and more adhesion of surrounding tissue was observed, which may be caused by inflammation. At the 8th week after injection treatment, the tendons in sham group seemed to be worse shaped (Supplementary Fig. 1D). These results showed better tendon structure and morphology in the TDSC-GM and PRP-TDSC-GM groups than in the SHAM group. Overall, tendons in all groups showed a shift from thick to slender, indicating that the tendon reshaped itself over time. In addition, obvious calcification was observed in the SHAM group in the eighth week. In the fourth week, significant angiogenesis developed in the PRP-TDSC-GM group, while in the eighth week, the blood vessels disappeared. VEGF and other cytokines in PRP could promote angiogenesis in the early stage after injection. Furthermore, PRP contains a variety of anti-inflammatory factors, which help with tissue remodelling. Therefore, neovascularization gradually disappeared, and the structure of the tendon was restored.

**Fig. 5:**
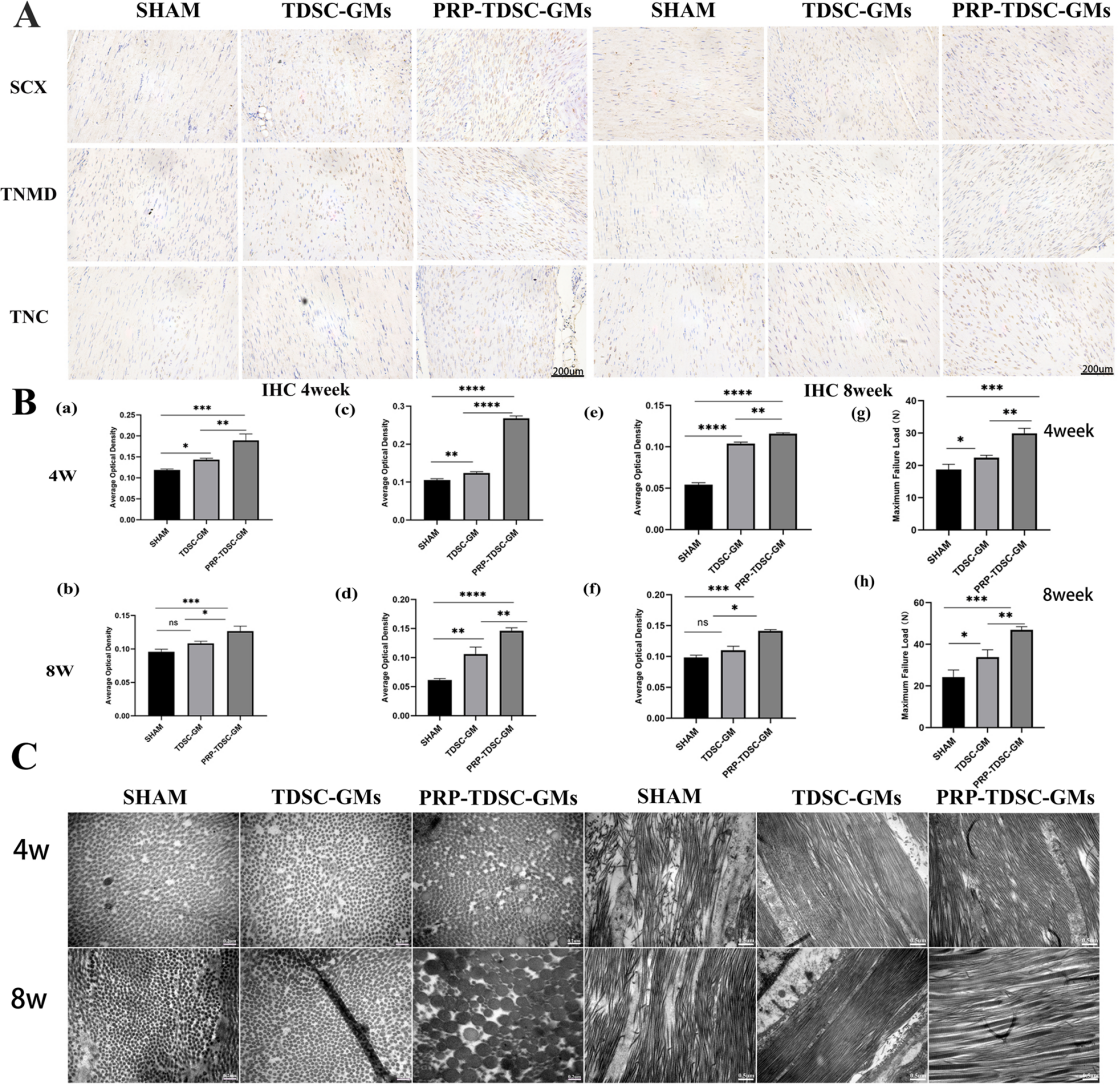
**A** Immunohistochemical images of tendinous protein staining in tendon samples from the different groups in the 4th week and 8th week after treatment. **B** (a–f) Quantitative analysis of IHC staining. (a, b) SCX expression. (c, d) TNMD expression. (e, f) TNC expression. (n = 3 *p < 0.05, **p < 0.01, ***p < 0.001). (g-h) Evaluation of the tension of tendons in the different groups (n = 3 *p < 0.05, **p < 0.01, ***p < 0.001). **C** Microstructure of tendon collagen fibres under a transmission electron microscope

After 4 weeks of treatment, immunohistochemical and immunofluorescence staining showed that the expression of SCX, TNMD and TNC in the PRP-TDSC-GM group was higher than that in the TDSC-GM and SHAM groups and the untreated group (Figs. 4C and F and 5A). After 8 weeks, the expression of three tendon-related proteins in the PRP-TDSC-GM group was higher than that in the other two groups. However, it is worth noting that in the 8th week, there was no significant difference in SCX and TNC protein expression between the TDSC-GM group and SHAM group, and only TNMD expression was upregulated (Figs. 4D and 5A). CD14 is thought to be specifically expressed by monocytes and macrophages, which are the main kinds of inflammatory cells during inflammatory reactions. During the 4th week and the 8th week after injection, more CD14^ +^ cells appeared in the SHAM group and TDSC-GM group than in the PRP-TDSC-GM group (Fig. 4E and G). We attributed this finding to the anti-inflammatory effects of PRP. However, during the 4th week, there were more CD14 ^+^ cells in the TDSC-GM group than in the SHAM group, but there was no difference between the two groups in the 8th week. We hypothesize that this may be related to the transient protective response of the body to exogenous substances.

### Biomechanical and TEM analysis of tendons

The subtle structural differences in the Achilles tendon between groups were examined by transmission electron microscopy. In the SHAM group, the collagen fibrils were messily organized with more tangles and fragments, and the gaps between fibres were uneven. In contrast, the collagen fibrils showed better arrangement and fewer clutters in the PRP-TDSC-GM group (Fig. 5C). The average diameter of collagen fibrils in the PRP-TDSC-GM group was significantly larger than that in the other two groups. The tensile mechanical properties were measured to evaluate the recovery of injured tendons. The maximum load of the PRP-TDSC-GM group was significantly higher than that of the SHAM group and TDSC-GM group (30 N at 4 weeks and 50 N at 8 weeks) (Fig. 5g, h).

### Inhibition of the PI3K-AKT axis by PRP-GM under IL-1 stimulation

**Fig. 6:**
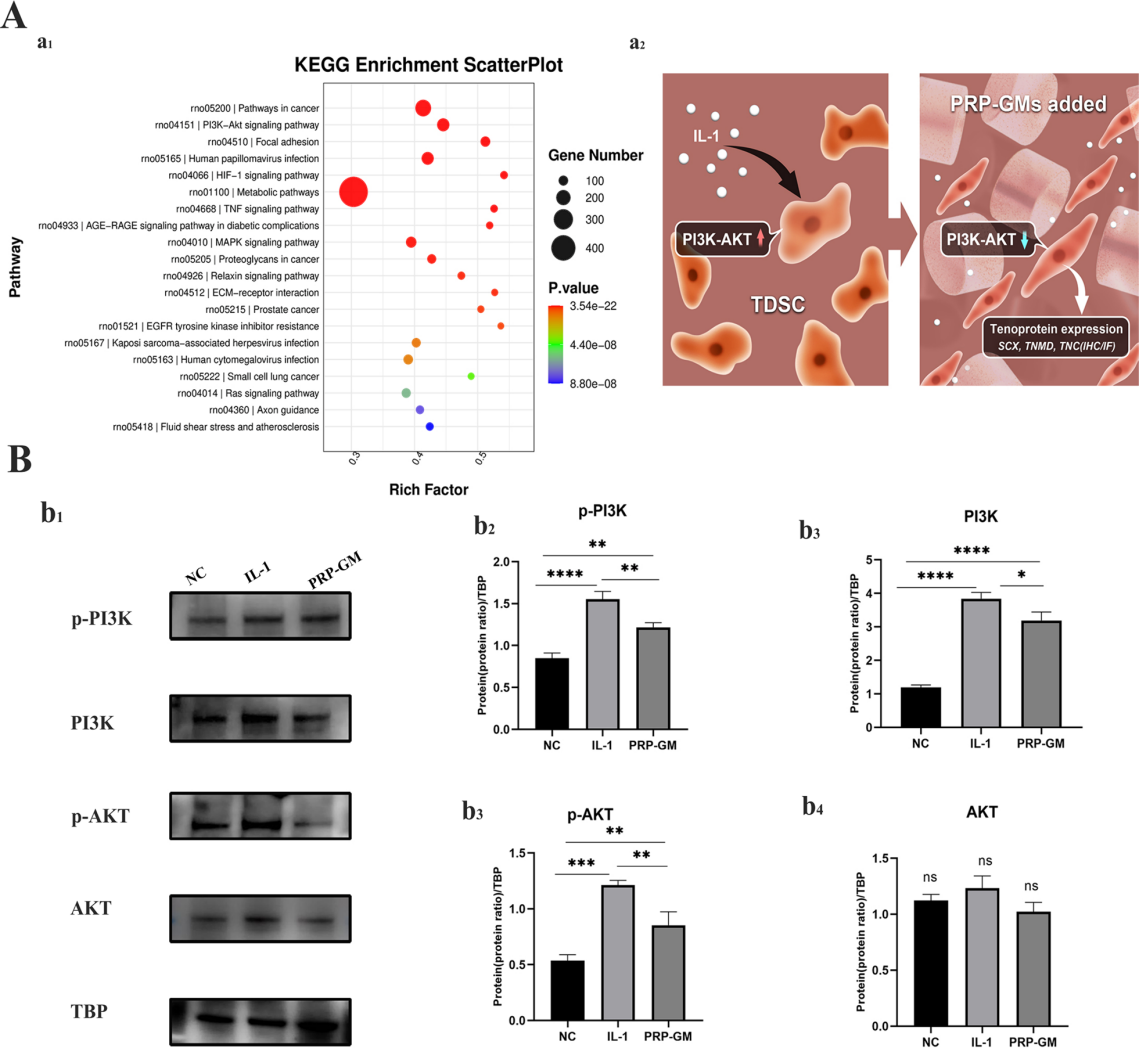
**A** (a_1_) Significantly enriched KEGG pathway terms. (a_2_) PRP-GM promotes tendon repair by downregulating the PI3K-AKT pathway. **B** Western blot analysis of p-PI3K, PI3K, p-AKT and AKT expression in TDSCs for 5 days in the different groups. (b_2_ -b_4_) Quantitative analysis of protein expression in the PI3K-AKT pathway (n = 3, *p < 0.05, **p < 0.01, *** p < 0.001)

After being cultured under different conditions for 3 days, transcriptional profiling of TDSCs in the different groups was analysed by RNA sequencing. Kyoto Encyclopaedia Gene and Genomes (KEGG) analysis indicated that the PI3K–AKT pathway was significantly activated in the pairwise comparison of the three groups (Fig. 6A). The Western blot results showed that the PI3K–AKT pathway was upregulated by stimulation with the cytokine IL-1 but downregulated in the presence of PRP-GM (Fig. 6B).

## Discussion

As a common therapy for treating tendinopathy, proper concentrations of PRP can promote tendon repair. However, cell proliferation and growth are inhibited by high concentrations of PRP. Therefore, a microgel containing PRP was designed for tendinopathy treatment. In this study, our experimental results showed that (1) PRP-GM could promote the migration and differentiation of tendon-derived stem cells and downregulate the PI3K-AKT pathway in an inflammatory environment in vitro; and (2) PRP-TDSC-GM could promote structural and functional repair of tendons in vivo.

GelMA is a gelatine-based engineering material with excellent biocompatibility and is easily biologically modified. This substance has been widely used for tissue engineering, drug delivery, cell culture and 3D bioprinting [34]. Amino groups in the side chain of gelatine are replaced by methacryloyl groups to form GelMA. In the presence of a photoinitiator, GelMA can form a porous gel under light irradiation, which makes it an excellent carrier for drugs, cytokines and cells. Additionally, gelatine is the hydrolysate of collagen and contains arginine-glycine-aspartic acid (RGD) sequences that promote cell attachment [35] and matrix metalloproteinase (MMP) target sequences suitable for cell remodelling [36]. Therefore, in this study, GelMA was selected as a carrier of PRP and TDSCs for the treatment of tendinopathy.

Currently, a series of approaches based on water-oil phase interactions have been developed to manufacture microgels, including electrospray, microfluidic chip and electrospinning [37]. Among these approaches, the loss of encapsulated drugs or cytokines is inevitable during the process of washing off the oil phase. In this study, by controlling the height of the printing platform and the appropriate modelling method, single-layer printing and molding of microgels could be realized, and repeated elution was avoided to protect the encapsulated factors. Moreover, during the traditional extrusion manufacturing process, the sprinkler can only produce one microsphere at a time. In the electrospray process, the size of the microspheres is strictly limited due to gravity. However, with the projection-based 3D bioprinting technique, through appropriate modelling and adjusting the specification of the printing platform, a large number of hydrogel particles of different sizes can be quickly produced at one time.

In tendons, TDSCs account for a small part (< 5%) but can renew themselves and differentiate into tendon cells and are responsible for tendon repair in the event of injury [38, 39]. TDSCs are known to take part in tendon homeostasis and repair [21, 23]. The direction of stem cell differentiation is often influenced by cell characteristics and the microenvironment. TDSCs may more closely resemble tendon cell phenotypes than other stem cells, such as BMSCs and ADSCs, potentially making them more suitable for tendon repair [40]. In addition, it has been reported that senescent TDSCs exhibit decreased tenogenic differentiation potential and proliferation by spreading senescence to surrounding cells during chronic inflammation [41].Therefore, it is critical to implant sufficient naive tendon stem cells and provide a suitable microenvironment to improve the possibility of their differentiation into tendon cells for tendon repair. It has been reported that platelet-rich plasma promotes the differentiation of tendon stem cells into active tenocytes [12, 42].The anti-inflammatory compounds in PRP also help to improve the microenvironment around cells. Giovanni et al. found that PRP can block bone deposition and increase the ratio of type III to type I collagen and stem cell chemotaxis [43]. PRP therapy has been proven to be effective in the clinic, but there is no consensus on the optimal concentration [44]. Previous studies have shown that the effects of PRP vary depending on its concentration [45]. In response to low-concentration PRP treatment, TDSCs gradually differentiate into slender tendon cells, while in response to high-concentration PRP treatment, TDSCs became square and sparse. When the concentration reached 20%, the growth of TDSCs was inhibited [46]. We hypothesized that the gelation of PRP after activation hinders the penetration of oxygen and nutrients, resulting in poor cell growth. In this study, PRP-GM did not form a gel after activation and promoted cell growth and differentiation in vitro.

Persistent chronic inflammation after acute tendon injury leads to poor healing and increases the risk of re-rupture [47, 48]. Increased CD14^+^ and CD68^+^ cells gather around the lesion and mediate complex inflammatory processes, including activation of the NF-κB, interferon and STAT-6 pathways [4]. Studies have shown that PRP may inhibit the release of IL-1 from activated macrophages [49], and HGF contained in PRP plays a major role in the anti-inflammatory process [13].

Additionally, the transcriptional sequencing results showed that there were significant differences in the PI3K-AKT pathway among the three groups. In general, the PI3K-AKT pathway mediates signals from multiple receptors, regulates inflammation and cell metabolism [50] and is important for cell survival, growth and proliferation [48]. Different opinions regarding the PI3K-AKT pathway have been proposed in recent years. Zhou Yiting et al. found that activating the AKT-mTOR pathway could promote the differentiation of TDSCs [51]. In the review by Chen et al., it was suggested that overexpression of PI3K-AKT was beneficial to the survival and differentiation of loaded cells in tissue engineering [52]. However, under long-term chronic inflammatory stimulation, the overactivation of this pathway may cause unnecessary negative effects. In other studies, inhibition of the PI3K-AKT pathway reduced the inflammatory response [53]. Cao et al. suggested that PRP pretreatment of tendon cells could downregulate PI3K-AKT-mTOR pathway to protect tendon cells from injury caused by hypoxia [54]. Xue et al. found that the inflammatory response during arthritis could be reduced by downregulating the PI3K-AKT-mTOR pathway. We suggest that at the early stage of tendon injury, the PI3K-AKT pathway is activated in response to stress to promote cell proliferation and begin early repair. During the late stage of tendon repair, inflammation continuously induces the PI3K-AKT pathway, and this overactivation attenuates tendon repair. In vitro, our results showed that PRP-GM inhibited the PI3K-AKT pathway during IL-1 stimulation, which was consistent with previous studies. Moreover, osteogenic differentiation of TDSCs mediated by inflammatory factors is considered to play a key role in calcific tendon disease. Previous studies have shown that the PI3K-AKT signalling pathway is essential in PGE2-induced osteogenic differentiation [55]. However, whether PRP-GM can attenuate tendon calcification through the PI3K-AKT pathway remains unclear. In this study, the addition of IL-1 to the medium could not completely simulate the complex inflammatory situations in vivo, suggesting that better modelling approaches are needed.

## Conclusions

Injectable PRP-GM was prepared by a projection-based 3D bioprinting technique. The bioactivation of the encapsuled factors was well protected by avoiding repeated elution and organic solvents in this system. PRP-GM showed a good biocompatibility profile for tendon repair by facilitating the proliferation and differentiation of TDSCs. This composite could help with treating chronic tendinopathy by downregulating the PI3K-AKT pathway in an inflammatory environment in vitro. However, further studies are required to determine its clinical potential in chronic tendinopathy.

## Supplementary Information

**Additional file 1: Figure S1.** A. KEGG analysis performed by pairwise comparison B. Rat model of tendinopathy and tension test machine C. Quantitative analysis of cell viability of TDSCs on GMs and PRP-GMs D. orthostatic and posterior -anterior macroscopic appearance of tendon. **Figure S2**. A. Differentiation of TDSCs outside the GMs B.Differentiation of TDSCs on the GMs C. PI3K-AKT pathway influenced by PRP-GMs.
