# Supplementary material for: 3D-printed hydrogel particles containing PRP laden with TDSCs promote tendon repair in a rat model of tendinopathy
Source: J Nanobiotechnology. 2023 Jun 3;21:177. doi: 10.1186/s12951-023-01892-5 (PMC10239155; doi:10.1186/s12951-023-01892-5)
Supplement: Supplementary file 1 — Additional file 1: Figure S1. A. KEGG analysis performed by pairwise comparison B. Rat model of tendinopathy and tension test machine C. Quantitative analysis of cell viability of TDSCs on GMs and PRP-GMs D. orthostatic and posterior -anterior macroscopic appearance of tendon. Figure S2. A. Differentiation of TDSCs outside the GMs B.Differentiation of TDSCs on the GMs C. PI3K-AKT pathway influenced by PRP-GMs. [file 12951_2023_1892_MOESM1_ESM.pdf]

Supplementary materials

Fig.1

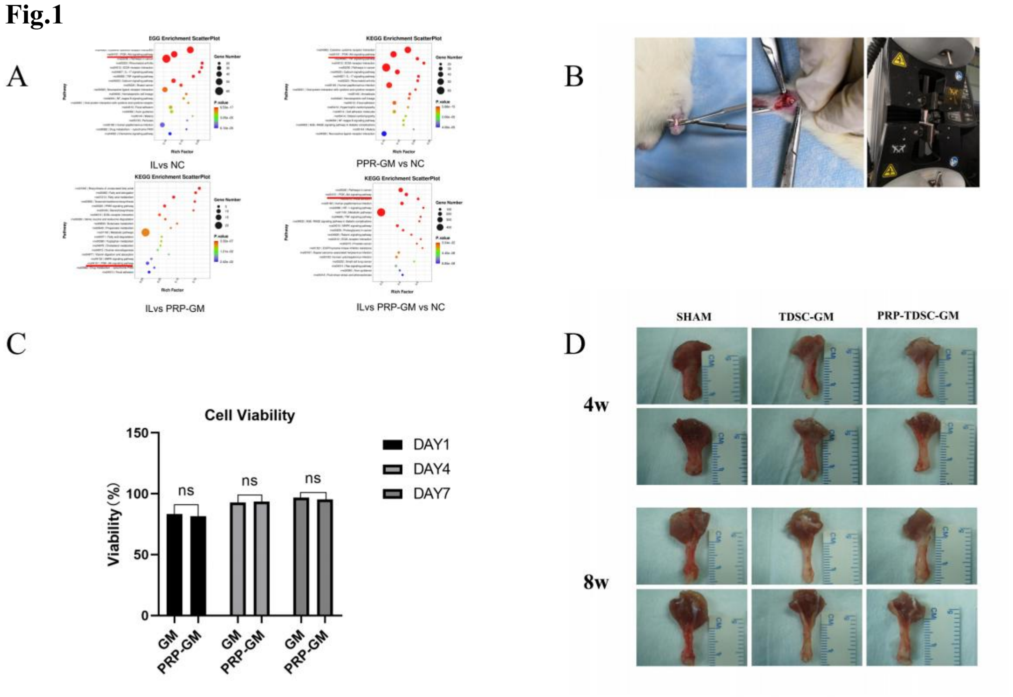

A. KEGG analysis performed by pairwise comparison B. Rat model of tendinopathy and tension test machine C. Quantitative analysis of cell viability of TDSCs on GMs and PRP-GMs D. orthostatic and posterior -anterior macroscopic appearance of tendon

Fig.2

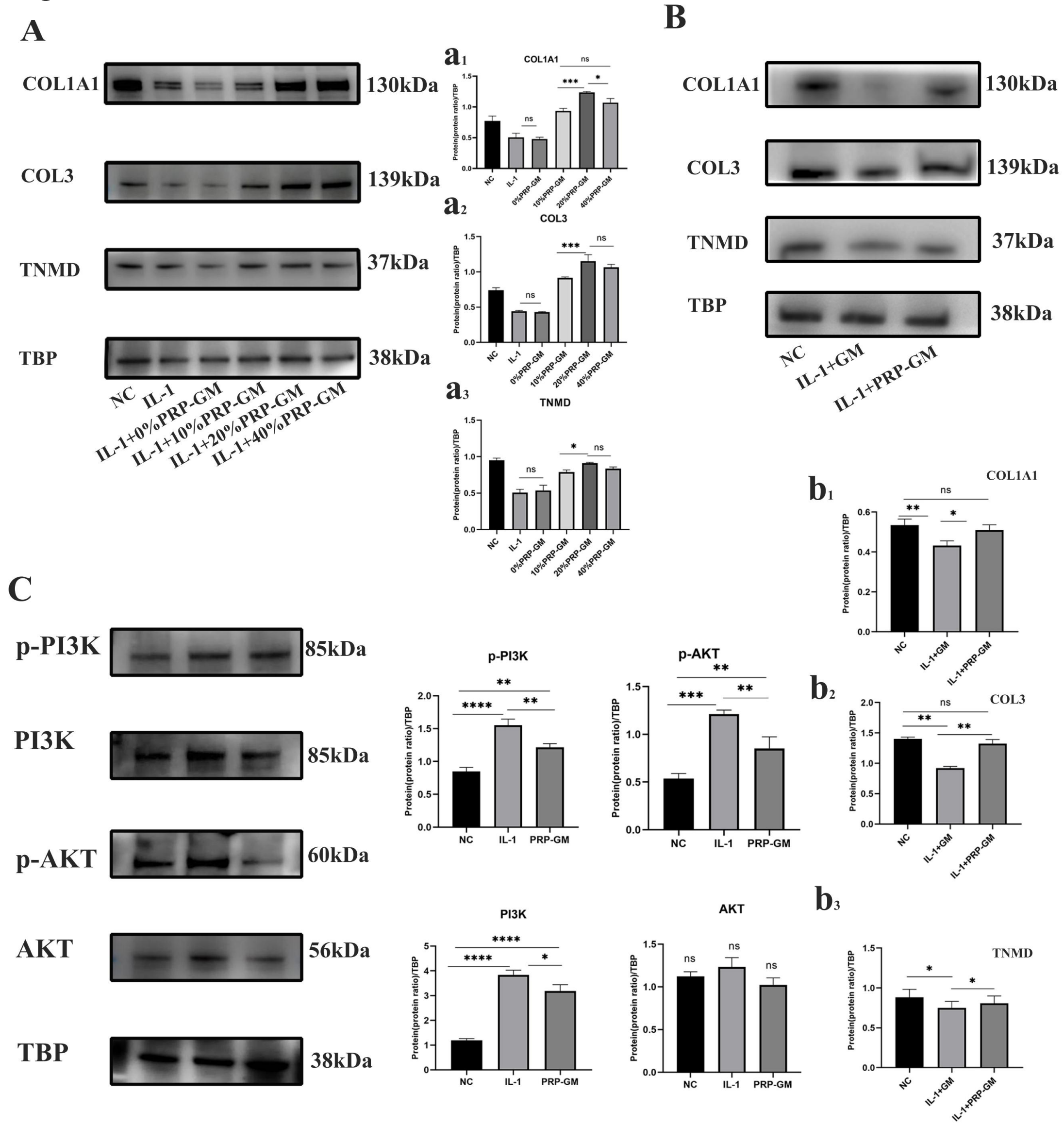

A.Differentiation of TDSCs outside the GMs B.Differentiation of TDSCs on the GMs C. PI3K-AKT pathway influenced by PRP-GMs
